# Supplementary material for: The Antimicrobial Susceptibility of Porphyromonas gingivalis: Genetic Repertoire, Global Phenotype, and Review of the Literature
Source: Antibiotics (Basel). 2021 Nov 24;10(12):1438. doi: 10.3390/antibiotics10121438 (PMC8698109; doi:10.3390/antibiotics10121438)
Supplement: Supplementary file 1 [file antibiotics-10-01438-s001.zip › antibiotics-1458952-supplementary.pdf]

Supplementary Material

Table S1. Strain information available for search on antimicrobial resistance genes.

Experiment accession	Sample title	Name of isolate	Clinical type	Isolation site	Geographical origin
DRX019659	Porphyromonas gingivalis ATCC 53977	ATCC53977	Periodontitis	Subgingival	USA
DRX019660	Porphyromonas gingivalis D3	D3	Periodontitis, acute	Subgingival	Japan
DRX019661	Porphyromonas gingivalis D4	D4	Periodontitis, acute	Subgingival	Japan
DRX019662	Porphyromonas gingivalis D5	D5	Periodontitis, acute	Subgingival	Japan
DRX019663	Porphyromonas gingivalis D8	D8	Periodontitis, acute	Subgingival	Japan
DRX019664	Porphyromonas gingivalis D9	D9	Periodontitis, acute	Subgingival	Japan
DRX019665	Porphyromonas gingivalis D12	D12	Periodontitis, aggressive	Subgingival	Japan
DRX019666	Porphyromonas gingivalis D26	D26	Periodontitis, aggressive	Subgingival	Japan
DRX019667	Porphyromonas gingivalis D14	D14	Periodontitis, acute	Subgingival	Japan
DRX019668	Porphyromonas gingivalis D15	D15	Periodontitis, acute	Subgingival	Japan
DRX019669	Porphyromonas gingivalis D16	D16	Periodontitis, acute	Subgingival	Japan
DRX019670	Porphyromonas gingivalis D17	D17	Periodontitis, acute	Subgingival	Japan
DRX019671	Porphyromonas gingivalis D18	D18	Periodontitis, acute	Subgingival	Japan
DRX019672	Porphyromonas gingivalis D19	D19	Periodontitis, acute	Subgingival	Japan
DRX019673	Porphyromonas gingivalis D22	D22	Periodontitis, acute	Subgingival	Japan
DRX019674	Porphyromonas gingivalis D23	D23	Periodontitis, acute	Subgingival	Japan
DRX019675	Porphyromonas gingivalis D28	D28	Periodontitis, acute	Subgingival	Japan
DRX019676	Porphyromonas gingivalis D29	D29	Periodontitis, acute	Subgingival	Japan
DRX019677	Porphyromonas gingivalis D45	D45	Periodontitis, acute	Subgingival	Japan
DRX019678	Porphyromonas gingivalis D32	D32	Periodontitis, acute	Subgingival	Japan
DRX019679	Porphyromonas gingivalis D33	D33	Periodontitis, acute	Subgingival	Japan
DRX019680	Porphyromonas gingivalis D34	D34	Periodontitis, acute	Subgingival	Japan
DRX019681	Porphyromonas gingivalis D39	D39	Periodontitis, acute	Subgingival	Japan
DRX019682	Porphyromonas gingivalis D40	D40	Periodontitis, acute	Subgingival	Japan
DRX019683	Porphyromonas gingivalis D41	D41	Periodontitis, acute	Subgingival	Japan
DRX019684	Porphyromonas gingivalis PC9	PC9	Periodontitis, acute	Subgingival	Japan
DRX019685	Porphyromonas gingivalis PC13	PC13	Periodontitis, acute	Subgingival	Japan
DRX019686	Porphyromonas gingivalis FK2	FK2	Periodontitis, acute	Subgingival	Japan
DRX019687	Porphyromonas gingivalis KS14	KS14	Periodontitis, aggressive	Subgingival	Japan
DRX019688	Porphyromonas gingivalis L1	L1	Periodontitis, acute	Subgingival	Japan
DRX019689	Porphyromonas gingivalis US4	US4	Periodontitis, acute	Subgingival	Japan
DRX019690	Porphyromonas gingivalis TDC59	TDC59	Periodontitis	Subgingival	Japan
DRX019691	Porphyromonas gingivalis TDC117	TDC117	Periodontitis	Subgingival	Japan
DRX019692	Porphyromonas gingivalis TDC129	TDC129	Periodontitis	Subgingival	Japan
DRX019693	Porphyromonas gingivalis TDC222	TDC222	Periodontitis	Subgingival	Japan
DRX019694	Porphyromonas gingivalis TDC225	TDC225	Periodontitis	Subgingival	Japan
DRX019695	Porphyromonas gingivalis TDC243	TDC243	Periodontitis	Subgingival	Japan
DRX019696	Porphyromonas gingivalis TDC260	TDC260	Periodontitis	Subgingival	Japan
DRX019697	Porphyromonas gingivalis TDC275	TDC275	Periodontitis	Subgingival	Japan
DRX019698	Porphyromonas gingivalis TDC280	TDC280	Periodontitis	Subgingival	Japan
DRX019699	Porphyromonas gingivalis HG184	HG184	NA	NA	NA
DRX019700	Porphyromonas gingivalis HG564	HG564	NA	NA	NA
DRX019701	Porphyromonas gingivalis HG1025	HG1025	NA	NA	NA
DRX019702	Porphyromonas gingivalis HW24D1	HW24D1	NA	NA	NA
DRX019703	Porphyromonas gingivalis ESO101	ESO101	NA	NA	NA
DRX019704	Porphyromonas gingivalis ESO132	ESO132	NA	NA	NA
DRX019705	Porphyromonas gingivalis OS30-2	OS30-2	NA	NA	NA
DRX019706	Porphyromonas gingivalis OS54-1	OS54-1	NA	NA	NA
DRX019707	Porphyromonas gingivalis OS61	OS61	NA	NA	NA
DRX019708	Porphyromonas gingivalis OMZ314	OMZ314	NA	NA	NA
DRX019709	Porphyromonas gingivalis Co5	Co5	Periodontitis, aggressive	Subgingival	Japan
DRX040827	Porphyromonas gingivalis	Ando	NA	NA	Japan
ERX1066718	Genomic DNA Sequence of the oral bacteria	3_3	NA	Subgingival pl	USA
ERX1066719	Genomic DNA Sequence of the oral bacteria	3A1	NA	Subgingival pl	Norway
ERX1066720	Genomic DNA Sequence of the oral bacteria	7BTORR	NA	Subgingival pl	USA
ERX1066721	Genomic DNA Sequence of the oral bacteria	11A	NA	Subgingival pl	Romania
ERX1066722	Genomic DNA Sequence of the oral bacteria	13_1	NA	Subgingival pl	Sudan
ERX1066723	Genomic DNA Sequence of the oral bacteria	15_9	NA	Subgingival pl	Romania
ERX1066724	Genomic DNA Sequence of the oral bacteria	84_3	NA	Subgingival pl	Sudan
ERX1066725	Genomic DNA Sequence of the oral bacteria	49417	NA	Periodontal p	Canada
ERX1066726	Genomic DNA Sequence of the oral bacteria	A7A1_28	NA	Subgingival pl	USA
ERX1066727	Genomic DNA Sequence of the oral bacteria	AFR5B1	NA	Subgingival pl	Sudan
ERX1066728	Genomic DNA Sequence of the oral bacteria	YH522	NA	Subgingival pl	Japan
ERX1066730	Genomic DNA Sequence of the oral bacteria	W50_ABK_WbaP	NA	Subgingival pl	Germany
ERX2022743	512915	512915	Periodontitis	Periodontitis	The Netherlands
ERX2022744	512919	512919	Periodontitis	Periodontitis	The Netherlands
ERX2022745	513125	513125	Periodontitis	Periodontitis	The Netherlands
ERX2022746	20663	20663	Periodontitis	Periodontitis	The Netherlands
ERX2022747	513324	513324	Periodontitis	Periodontitis	The Netherlands
ERX2022748	513044	513044	Periodontitis	Periodontitis	The Netherlands
ERX2022749	505759	505759	Periodontitis	Periodontitis	The Netherlands
ERX2022750	MDS33	MDS33	Periodontitis	Periodontitis	The Netherlands
ERX2022751	515430	515430	Periodontitis	Periodontitis	The Netherlands
ERX2069209	19X2-K1	19X2-K1	Periodontitis	Periodontitis	The Netherlands
SRX1437042	PgW50-NIDCR	PgW50-NIDCR	NA	NA	USA
SRX1437565	PgW50-ATCC	PgW50-ATCC	NA	NA	USA
SRX7089586	ATCC33277	ATCC33277	Gingival sulcus	NA	NA
